# Concurrent chemoradiotherapy with S-1 compared with concurrent chemoradiotherapy with docetaxel and cisplatin for locally advanced esophageal squamous cell carcinoma

**DOI:** 10.1186/s13014-021-01821-6

**Published:** 2021-05-26

**Authors:** Xi-Lei Zhou, Chang-Hua Yu, Wan-Wei Wang, Fu-Zhi Ji, Yao-Zu Xiong, Wei-Guo Zhu, Yu-Suo Tong

**Affiliations:** Department of Radiation Oncology, Huai’an First People’s Hospital, Nanjing Medical University, Huai’an, Jiangsu China

**Keywords:** Esophageal cancer, Chemoradiotherapy, S-1, Docetaxel, Cisplatin

## Abstract

**Background:**

This retrospective study was to assess and compare the toxicity and efficacy of concurrent chemoradiotherapy (CCRT) with S-1 or docetaxel and cisplatin in patients with locally advanced esophageal squamous cell carcinoma (ESCC).

**Methods:**

Patients with locally advanced ESCC who received CCRT with S-1 (70 mg/m^2^ twice daily on days 1–14, every 3 weeks for 2 cycles, S-1 group) or docetaxel (25 mg/m^2^) and cisplatin (25 mg/m^2^) on day 1 weekly (DP group) between 2014 and 2016 were retrospectively analyzed. Radiotherapy was delivered in 1.8–2.0 Gy per fraction to a total dose of 50–60 Gy. Treatment-related toxicities (Common Terminology Criteria for Adverse Events version 4.0), response rate, and survival outcomes were compared between groups.

**Results:**

A total of 175 patients were included in this study (72 in the S-1 group and 103 in the DP group). Baseline characteristics were well balanced between the two groups. The incidence of grade 3–4 adverse events were significantly lower in the S-1 group than that of the DP group (22.2% vs. 45.6%, *p* = 0.002). In the DP group, elderly patients (> 60 years) had a significantly higher rate of grade 3–4 adverse events than younger patients (58.1% vs. 31.3%, *p* = 0.01). The objective overall response rate (complete response + partial response) was 68.1% in the S-1 group, and 73.8% the DP group (*p* = 0.497). The 3-year overall survival was 34.7% in the S-1 group, and 38.8% in the DP group (*p* = 0.422). The 3-year progression free survival in the DP group was higher than that in the S-1 group but without significant difference (33.0% vs. 25.0%, *p* = 0.275).

**Conclusion:**

CCRT with S-1 is not inferior to CCRT with docetaxel and cisplatin and is better tolerated in in elderly patients with locally advanced ESCC.

**Supplementary Information:**

The online version contains supplementary material available at 10.1186/s13014-021-01821-6.

## Background

Esophageal squamous cell cancer (ESCC) is the fourth leading cause of cancer-related death in China [1]. Approximate 70% of patients with ESCC have locally advanced diseases [2]. Although, concurrent chemoradiotherapy (CCRT) has been regarded as the standard treatment [3], the optimal concurrent chemotherapy regimen remains controversial. The combination of cisplatin and fluorouracil (PF) is one of the most commonly used concurrent chemotherapy regimens [4]. In Radiation Therapy Oncology Group (RTOG) 85-01, concurrent chemotherapy could be administered in only 89 of 130 patients (68%) because of PF-related toxic effects. Furthermore, even after CCRT, long-term survival remains unsatisfactory, with a 5-year OS of less than 27% [5]. In attempt to improve prognosis, many chemotherapeutic agents have been evaluated, such as docetaxel, paclitaxel, and cetuximab [6]. Of them, the combination of docetaxel with cisplatin has shown promising results in patients with locally advanced or recurrent ESCC when combined with radiotherapy (RT) [7, 8]. A recent randomized trial demonstrated that this regimen could result in higher overall response rates and longer OS compared with PF-based CCRT with tolerable toxicities in locally advanced ESCC [9].

S-1, a new biochemical modulator of 5-fluorouracil, is an oral fluorinated pyrimidine formulation composed of tegafur (a prodrug of 5-FU), gimeracil, and oteracil [10]. The anticancer effect of S-1 has been demonstrated in gastric cancer, non-small cell lung cancer, head and neck cancer, and pancreatic cancer [11–13]. In addition to cytotoxic activity, S-1 can enhance radiosensitivity in tumor cells by suppressing Akt/PKB activation [14]. In a phase I/II trial for stage II/III ESCC, chemoradiotherapy with concurrent S-1 and cisplatin showed highly promising activity with a complete response rate of 59.5% [15]. Although, a number of studies have demonstrated encouraging results of S-1 in ESCC treatment [16–18], no study has investigated the safety and efficacy of docetaxel plus cisplatin versus S-1 concurrent with RT in patients with ESCC.

The aim of this study was to compare the differences in toxicity rates and efficacy between S-1 and docetaxel with cisplatin as CCRT in patients with locally advanced ESCC.

## Methods

### Patients

This study was approved by the institutional review board of Huai’an First Hospital. Informed consent was exempted due to the retrospective nature of the study. Between January 2014 and December 2016, patients with locally advanced ESCC (UICC, 6th edition) who received CCRT with S-1 or docetaxel and cisplatin at our institute were retrospectively analyzed [19]. Inclusion criteria were histologically confirmed ESCC, Karnofsky performance status (KPS) score ≥ 70, less than 75 years old, previously untreated, and no severely abnormal cardiac, pulmonary, renal, or hepatic function. Patients with history of previous or concomitant malignancy were excluded as well as cases with missing relevant staging information or inadequate follow up. Clinical staging evaluations were based on esophagogram and enhanced CT scan of neck, chest and upper abdomen. Clinical data collected from each patient included age, gender, KPS score, primary tumor location, clinical stage, radiation dose, and tumor response to treatment. Blood samples at baseline, during (weekly) and 4 weeks after CCRT were also collected for evaluation haemoglobin concentration, neutrophil counts and platelet counts.

### Chemotherapy regimens

Chemotherapy began on day 1, concurrent with the beginning of RT.

S-1 group: S-1 (70 mg/m^2^) was given orally twice daily on days 1–14 every 3 weeks for 2 cycles. After CCRT, patients received two cycles of consolidation chemotherapy with S-1 at the same dose levels as during CCRT every 3 weeks.

Docetaxel + cisplatin (DP) group: The DP regimen comprised 5 or 6 cycles of docetaxel (25 mg/m^2^ per day) and cisplatin (25 mg/m^2^ per day) during RT at day 1, 8, 15, 22, 29, and 36. Four weeks after completion of CCRT, patients received 2 cycles of consolidation chemotherapy (docetaxel 75 mg/m^2^ on day 1, cisplatin 25 mg/m^2^ on days 1–3) with a 4-week interval.

### Radiotherapy

All of the patients underwent three-dimensional conformal radiotherapy (3D-CRT) or intensity modulated radiotherapy (IMRT) via a 6 MV X-ray beam. A total radiation dose of 50–60 Gy (median dose 50.4 Gy) was given in daily fractions of 1.8–2.0 Gy. The clinical target volume (CTV) included the gross tumor volume (GTV) with 3-cm craniocaudal margin, the metastatic lymph nodes, and regional lymph node. We have previously reported the details of regional lymph node for tumor in different locations at our institute [20].

### Toxicity and response assessment

In our center, acute treatment-related toxicity was monitored throughout the treatment and 3 months after CCRT. The hematology and biochemistry assessments were done every week during treatment and every month after the end of treatment. Toxicities were graded according to the National Institute Common Terminology Criteria for Adverse Events (NCI CTCAE) version 4.0.

The tumor responses (primary tumor and metastatic lymph node) were evaluated using the Response Evaluation Criteria in Solid Tumors (RECIST, Version 1.0) with esophagography and chest CT scan 4–6 weeks after completion of CCRT [21]. The objective response rate (ORR) was defined as complete response (CR) + partial response (PR).

### Follow-up evaluation

The first follow-up evaluation was performed 4–6 weeks after completion of CCRT, followed by every 6 months for the next 2 year, and then every year or until death. Follow-up data were obtained from patient medical records, referring physicians, and telephone interview. Each visit included patient history, physical examination, esophagogram and enhanced CT scan of neck, chest and upper abdomen. We evaluated post-treatment recurrence on esophagogram and CT scan and compared these data with original CT-based radiation treatment plan.

### Statistical analysis

Patient characteristics and treatment-related toxicity rates were compared using Chi-square test or Fisher’s exact test. OS was calculated from the date of diagnosis to death or last follow-up. Data from patients that had not died by the time of analysis were censored. Progression free survival (PFS) was defined as the time from diagnosis to first recurrence or metastasis, or last follow-up. Data from patients that were alive without tumor progression at the time of analysis were censored. Kaplan–Meier method was used to analyze survival. All analyses were performed using SPSS 20.0. A two-side *p* value less than 0.05 was considered statistically significant.

## Results

### Patient and treatment characteristics

A total of 175 patients (72 in the S-1 group and 103 in the DP group) were eventually included in this study. The baseline patient and tumor characteristics (location, tumor length, and clinical stage) were well balanced between the two groups. The median age was 64 years (range 47–74 years) in the S-1 group and 62 years (range 49–74 years) in the DP group. Patient and treatment characteristics are shown in Table 1.

**Table 1:** Baseline clinical characteristics

Patient characteristic	DP group (n = 103)	S-1 group (n = 72)	*p* value
Age (median, year)	62	64	0.647
< 60	48 (46.6%)	31 (43.1%)	
≥ 60	55 (53.4%)	41 (56.9%)	
*Gender*			0.873
Male	65 (63.1%)	47 (65.3%)	
Female	38 (36.9%)	25 (34.7%)	
*KPS*			0.756
90	43 (41.7%)	28 (38.9%)	
≥ 70	60 (58.3%)	44 (61.1%)	
*Tumor location*			0.394
Upper third	28 (27.2%)	16 (22.2%)	
Middle third	60 (58.3%)	40 (55.6%)	
Lower third	15 (14.5%)	16 (22.2%)	
*Primary tumor length (cm)*			0.759
< 5	56 (54.4%)	41 (56.9%)	
≥ 5	47 (45.6%)	31 (43.1%)	
*Smoking*			0.273
Nonsmoker	43 (41.7%)	24 (33.3%)	
Smoker	60 (58.3%)	48 (66.7%)	
*Drinking*			0.358
No	49 (47.6%)	29 (40.3%)	
Yes	54 (52.4%)	43 (59.7%)	
*Differentiation*			0.838
Well	14 (13.6%)	8 (11.1%)	
Moderate	59 (57.3%)	44 (61.1%)	
Poor	30 (29.1%)	20 (27.7%)	
*Clinical T stage*			1.000
No invasion to adjacent organs	56 (54.4%)	39 (54.2%)	
Invasion to adjacent organs	47 (45.6%)	33 (45.8%)	
*Clinical N stage*			0.867
N0	30 (29.1%)	20 (27.8%)	
N1	73 (70.9%)	52 (72.2%)	
*Reason for no surgery*			0.924
Patient refusal	8 (7.7%)	6 (8.3%)	
Surgical contraindication	15 (14.6%)	9 (12.5%)	
Unresectable disease	65 (63.1%)	44 (61.1%)	
Unknown	15 (14.6%)	13 (18.1%)	
*Radiation dose (Gy)*			0.698
50	34 (33.0%)	23 (31.9%)	
50.4	46 (44.7%)	29 (40.3%)	
> 50.4	23 (22.3%)	20 (27.8%)	
*Radiotherapy techniques*			0.410
3D-CRT	30 (29.1%)	26 (36.1%)	
IMRT	73 (70.9%)	46 (63.9%)	
*Consolidation chemotherapy*			0.537
Yes	61 (59.2%)	39 (54.2%)	
No	42 (40.8%)	33 (45.8%)	
*Nutritional support*			0.265
Yes	17 (16.5%)	7 (9.7%)	
No	86 (83.5%)	65 (90.3%)	

*KPS* Karnofsky performance status, *3D-CRT* three-dimensional conformal radiotherapy, *IMRT* intensity modulated radiotherapy

Of the treatment characteristics, 62 patients (86.1%) in the S-1 group completed the planned 2 cycles of S-1 based concurrent chemotherapy, while 73 patients (70.9%) in the DP group completed the all cycles of concurrent chemotherapy (*p* = 0.027). Planned RT was completed in 68 patients (94.4%) in S-1 group, and 94 patients (91.3%) in the DP group. In the S-1 group, 39 patients (54.2%) received 2 additional cycles of consolidation chemotherapy after CCRT, as did 61 patients (59.2%) in the DP group (*p* = 0.537).

### Treatment-related toxicities and mortality

Acute treatment-related toxicities are shown in Table 2. The incidence of grade 3–4 adverse events were significantly lower in the S-1 group than in the DP group (22.2% vs. 45.6%, *p* = 0.002). In the S-1 group, the most frequent grade 3–4 adverse events (≥ 5%) included leukopenia (12.5%), neutropenia (9.7%), anemia (5.6%), esophagitis (16.7%), and fatigue (6.9%). However, in the DP group, grade 3–4 adverse events included leukopenia (34.0%), neutropenia (29.1%), anemia (6.8%), thrombocytopenia (5.8%), esophagitis (23.3%), Nausea/vomiting (9.7%) and fatigue (8.7%). Grade 3–4 leukopenia and neutropenia were more frequently observed in the DP group than in the S-1 group (leukopenia: 34.0% vs. 12.5%, *p* = 0.001, neutropenia: 29.1% vs. 9.7%, *p* = 0.002). There were no statistical differences in the incidence of grade 3–4 non-hematological toxicities during CCRT between the two groups, with the exception of nausea/vomiting (9.7% vs. 1.4%, *p* = 0.028), which was more frequent in the DP group. There were 2 treatment-related deaths in the DP group. One patient died due to aspiration pneumonia. The other patient died due to trachea-esophageal fistula. No treatment-related death was observed in the S-1 group.

**Table 2:** Treatment-related toxicities during CCRT

Toxicities	DP group (n = 103)	S-1 group (n = 72)	*p* value
Overall toxicity ≥ 3	47 (45.6%)	16 (22.2%)	0.002
Grade 3	31 (30.1%)	13 (18.1%)	0.079
Grade 4	16 (15.5%)	3 (4.1%)	0.024
*Hematological toxicities ≥ 3*			
Anemia	7 (6.8%)	4 (5.6%)	0.767
Leukopenia	35 (34.0%)	9 (12.5%)	0.001
Neutropenia	30 (29.1%)	7 (9.7%)	0.002
Thrombocytopenia	6 (5.8%)	2 (2.8%)	0.473
*Non-hematological toxicities ≥ 3*			
Esophagitis	24 (23.3%)	12 (16.7%)	0.344
Nausea/vomiting	10 (9.7%)	1 (1.4%)	0.028
Mucositis	4 (3.9%)	3 (4.1%)	1.000
Fatigue	9 (8.7%)	5 (6.9%)	0.781
Pneumonitis	5 (4.9%)	3 (4.1%)	1.000

Next, we further investigated the adverse effects in elderly patients (Additional file 1: Table S1). A total of 96 patients (41 in the S-1 group and 55 in the DP group) were older than 60 years. In the S-1 group, the rate of ≥ grade 3 adverse effects was 24.3% (10/41) for patients > 60 years and 19.4% (6/31) for those ≤ 60 years (*p* = 0.776). However, in the DP group, the rate of ≥ grade 3 adverse effects was 58.1% (32/55) for patients > 60 years, which was significantly higher than those ≤ 60 years (31.3%, 15/48) (*p* = 0.01).

### Response to treatment

Tumor response is shown in Table 3. Of the 72 patients in the S-1 group, CR and PR were achieved in 18 patients (25.0%) and 31 patients (43.1%), respectively. In the DP group, 29 patients (28.2%) had a CR and 47 patients (45.6%) had a PR. The ORR was comparable between groups (68.1% vs. 73.8%, *p* = 0.497).

**Table 3:** Tumor response after treatment

Response	DP group n = 103	S-1 group n = 72	*p* value
CR	29 (28.2%)	18 (25.0%)	
PR	47 (45.6%)	31 (43.1%)	
SD	25 (24.3%)	21 (29.1%)	
PD	2 (1.9%)	2 (2.8%)	
ORR	76 (73.8%)	49 (68.1%)	0.497

*CR* complete response, *PR* partial response, *SD* stable disease, *PD* progressive disease, *ORR* objective response rate

### Follow up and survival

**Fig. 1:**
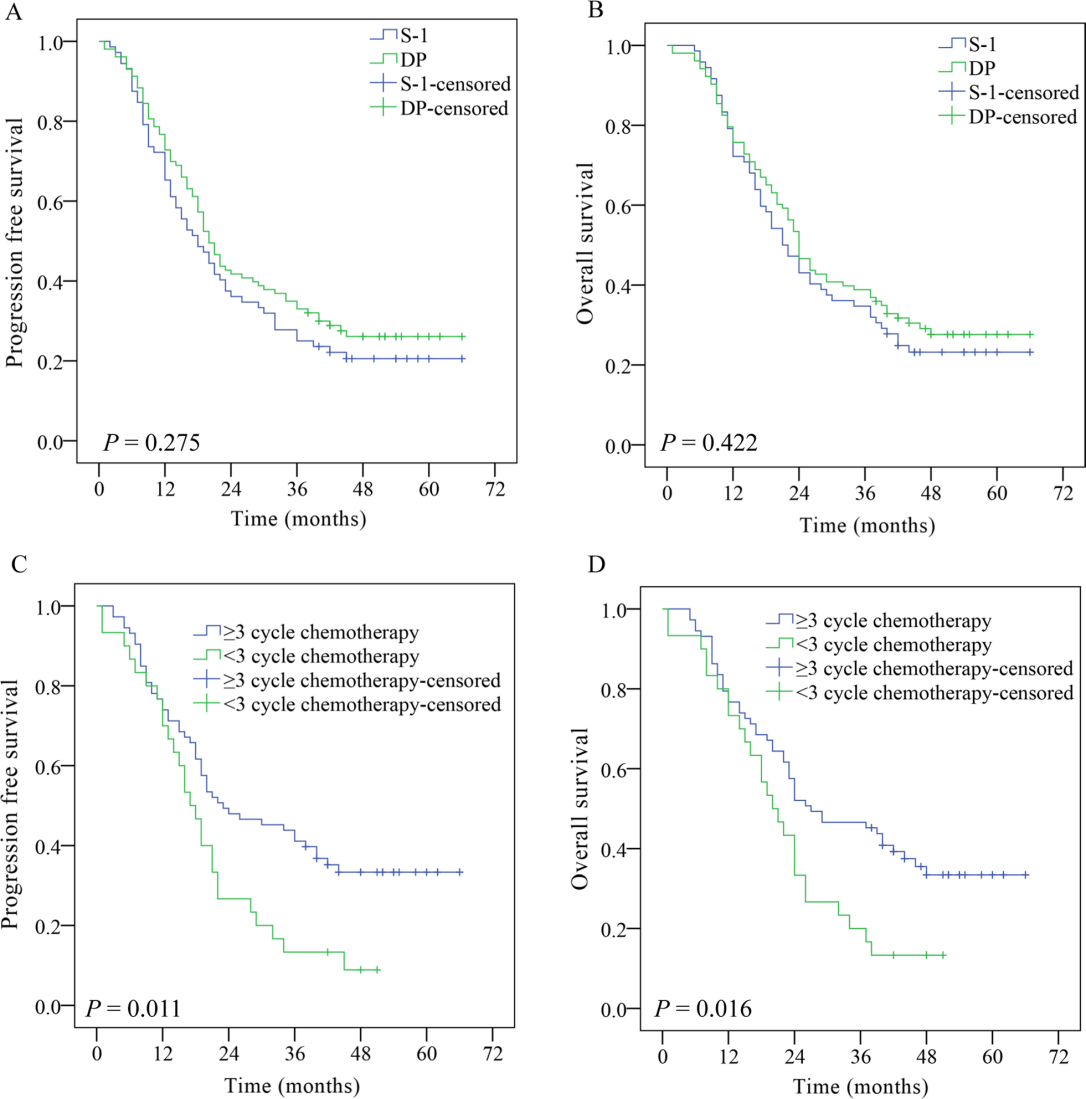
Kaplan–Meier estimates of progression free survival (**a**) and overall survival (**b**) in the S-1 and DP groups. Kaplan–Meier estimates by completeness of concurrent chemotherapy for progression free survival (**c**) and overall survival (**d**) using patients in the DP group

During the follow-up period, 132 patients experienced disease progression, with 57 patients (79.2%) in the S-1 group and 75 patients (72.8%) in the DP group. The 1- and 3-year PFS rates were 65.3% and 25.0%, respectively in the S-1 group, and 72.8% and 33.0%, respectively in the DP group. PFS was longer in the DP group than that in the S-1 group but without significant differences (Fig. 1a, p = 0.275). Of the 132 patients who had recurrence, 42.1% (24/57) of patients in the S-1 group and 32.0% (24/75) of patients in the DP group received targeted therapy or PD-1 inhibitor.

The median follow-up time was 19 months (range: 1–66 months). As of May 31, 2020, 55 patients (76.4%) in the S-1 group and 73 patients (70.9%) in the DP group had died or were followed for more than 3 years. The median OS was not reached in both groups. The 1- and 3-year survival rates were 72.2% and 34.7%, respectively in S-1 group, and 75.7% and 38.8%, respectively in DP group. As shown in Fig. 1b, OS was not significantly different between the two groups (*p* = 0.422).

Because there were more patients in the DP group who did not complete the all cycles of concurrent chemotherapy than in the S-1 group, we further investigated the role on survival. As shown in Fig. 1c, d, patients in the DP group who completed the planned concurrent chemotherapy had longer PFS (p = 0.011) and OS (*p* = 0.016) than those who did not.

## Discussion

In this retrospective study, we compared the clinical safety and efficacy between S-1 and DP as part of CCRT in patients with locally advanced ESCC. Our results showed that compared with DP, S-1 was non-inferior in terms of treatment response rate, PFS and OS. However, the incidences of severe treatment-related toxicities were significantly lower in S-1 group than in the DP group.

Concurrent chemotherapy with RT has been established as the standard treatment for patients with locally advanced ESCC [22]. The concurrent chemotherapy with PF is most commonly used in clinical practice. A recent phase II/III clinical trial, using PF-based CCRT, reported the ORR rate in 62% of ESCC patients and 7.5% survived for 3 years. In this study, the ORR and the 3-year OS were comparable or even better than those patients treated with PF-based CCRT [23].

Docetaxel is semi-synthetic taxanes that has radiosensitizing effect through inducing the G2/M cell cycle blockage [24]. Several recent clinical trials of docetaxel plus cisplatin concurrent with RT in locally advanced ESCC have shown promising results and manageable toxicity. In a phase II clinical trial, Shim Hj et al. evaluated CCRT with weekly DP for advanced ESCC, and showed that this regimen was convenient and well tolerable. The study reported the overall response rate of 85.8%, 3-year PFS of 16.7%, and 3-year OS of 27.8% [25]. In our study, the ORR was 73.8%, and the 3-year OS was 38.8% in the DP group, which is similar to the results of previously reported. In another phrase II clinical trial, Zhao et al. compared the CCRT with either DP or PF in patients with ESCC, and demonstrated a survival advantage from DP regimen [26]. In contrast, Zhu Y et al. compared with DP with PF in 86 patients with ESCC who received CCRT and showed that OS, PFS, and treatment response were nearly equivalent in both groups. However, the incidence of Grade ≥ 3 neutropenia was significantly higher in the DP-based treatment (68.9% vs. 19.5%) [27].

Compared with 5-fluorouracil, S-1, a fourth-generation orally active fluoropyrimidine, exhibits higher anti-cancer activity and lower side effect. In a retrospective analysis of CCRT with S-1 in 68 patients with stage I–IV ESCC, the median OS was 25.7 months, and the 1-, 3-, and 5-year OS were 70.6%, 41.8% and 25.9%, respectively [28]. In a prospective study of 30 patients received CCRT with S-1 (70 mg/m^2^), the 2-year PFS and OS was 40.8% and 45.1%, respectively [29]. However, no study has directly compared the efficacy and safety of concurrent DP and S-1 regimens combined with RT in ESCC. In this study, the 3-year OS and PFS in the S-1 group was 34.7% and 25.0%, respectively. Compared with S-1, DP-based CCRT did not prolong PFS and OS. The non-significant difference in prognosis between the two groups may be due to the poor treatment completion rate in the DP group. In our study, only 73 patients (70.9%) in the DP group completed the concurrent chemotherapy as planned, which was significantly lower than that in the S-1 group (86.1%). The 3-year PFS and OS were significantly longer for patients who completed the planned concurrent chemotherapy than those who did not. Another reason could partially attribute to the therapy followed the study. More patients in the S-1 group (42.1% vs. 32.0%) received targeted therapy or PD-1 inhibitor after tumor progression.

Severe hematological toxicities are the main reason for treatment interruption or termination. In the present study, the incidences of grade 3–4 hematological toxicities were significantly higher in the DP group than in the S-1 group. The most frequent ≥ grade 3 toxicity in DP group was leukopenia, observed approximately in 34.0% of patients, which was higher than in previous studies [30]. Though analysis, we found that the higher adverse event rate may be explained by malnutrition (BMI ≤ 18.5 kg/m^2^, data not shown) and older age (> 60 years) before treatment. As most grade 3–4 leukopenia were observed at cycle 3, primary prophylactic granulocyte colony-stimulating factors (G-CSF) should be strongly considered in the management of these patients. In addition, several non-hematological toxicities (nausea and vomiting) were more frequent and severe in the DP group than in the S-1 group, most likely due to the use of highly emetogenic cisplatin. In this study, grade 3–4 adverse events were less observed in the S-1 group, with a rate of only 22.2%, which is concordant with that in Lv S’s report [28]. CCRT with S-1 means a lower incidence of treatment-related toxicities, so this regimen should be more recommended for those who are fragile or older.

This study is limited for the short follow-up period and the retrospective design. Therefore, further multi-institutional prospective clinical trials are needed to confirm our results. In addition, the sample size was small and a larger sample would be needed to make these conclusions more robust.

## Conclusion

Our findings suggest that CCRT with S-1 is non-inferior to CCRT with DP in term of treatment response, PFS, and OS. CCRT with S-1 could be a new treatment option for patients with locally advanced ESCC, especially for patients aged over 60 years. Considering that potential bias may exist in this study, randomized clinical trials are needed to confirm these findings.

## Supplementary Information

**Additional file 1: Table S1.** Treatment-related toxicities in patients > 60 years.

## Data Availability

All data included in the present study were presented in the main manuscript.

## Abbreviations

CCRT: Concurrent chemoradiotherapy
ESCC: Esophageal squamous cell carcinoma
OS: Overall survival
PF: Cisplatin and fluorouracil
DP: Docetaxel and cisplatin
RT: Radiotherapy
KPS: Karnofsky performance status
3D-CRT: Three-dimensional conformal radiotherapy
IMRT: Intensity modulated radiotherapy
CTV: Clinical target volume
GTV: Gross tumor volume
NCI CTCAE: National Institute Common Terminology Criteria for Adverse Events
RECIST: Response Evaluation Criteria in Solid Tumor
ORR: Objective response rate
CR: Complete response
PR: Partial response
SD: Stable disease
PD: Progressive disease
PFS: Progression free survival
