# Supplementary material for: Concurrent chemoradiotherapy with S-1 compared with concurrent chemoradiotherapy with docetaxel and cisplatin for locally advanced esophageal squamous cell carcinoma
Source: Radiat Oncol. 2021 May 26;16:94. doi: 10.1186/s13014-021-01821-6 (PMC8157673; doi:10.1186/s13014-021-01821-6)
Supplement: Supplementary file 1 — Additional file 1: Table S1. Treatment-related toxicities in patients > 60 years. [file 13014_2021_1821_MOESM1_ESM.docx]

Additional file 1.

Table S1 Treatment-related toxicities in patients > 60 years

| Toxicities | DP group  (n=55) | S-1 group  (n=41) |
| --- | --- | --- |
| Hematological toxicities ≥3 |  |  |
| Anemia | 5 (9.1%) | 3 (7.3%) |
| Leukopenia | 16 (29.1%) | 4 (9.8%) |
| Neutropenia | 14 (25.5%) | 3 (7.3%) |
| Thrombocytopenia | 3 (5.5%) | 1 (2.4%) |
| Non-hematological toxicities ≥3 |  |  |
| Esophagitis | 10 (18.2%) | 7 (17.1%) |
| Nausea/vomiting | 5 (9.1%) | 1 (2.4%) |
| Mucositis | 1 (1.8%) | 2 (4.9%) |
| Fatigue | 4 (7.3%) | 1 (2.4%) |
| Pneumonitis | 1 (1.8%) | 2 (4.9%) |
